# Investigation of Microbial Community of Korean Soy Sauce (*Ganjang*) Using Shotgun Metagenomic Sequencing and Its Relationship with Sensory Characteristics

**DOI:** 10.3390/microorganisms12122559

**Published:** 2024-12-12

**Authors:** Young-Wook Chin, Sang-Pil Hong, Sang-Dong Lim, Sung-Hun Yi

**Affiliations:** Traditional Food Research Group, Korea Food Research Institute, Iseo-myeon, Wanju-gun 55365, Jeollabuk-do, Republic of Korea; sphong@kfri.re.kr (S.-P.H.); sdlim@kfri.re.kr (S.-D.L.); sunghunyi@kfri.re.kr (S.-H.Y.)

**Keywords:** metagenome, soy sauce, *ganjang*, microbial communities, sensory characteristics

## Abstract

The microbial community of a soy sauce is one of the most important factors in determining the sensory characteristics of that soy sauce. In this study, the microbial communities and sensory characteristics of twenty samples of Korean soy sauce (*ganjang*) were investigated using shotgun metagenome sequencing and descriptive sensory analysis, and their correlations were explored by partial least square (PLS) regression analysis. The metagenome analysis identified 1332 species of bacteria, yeasts, molds, and viruses across 278 genera, of which *Tetragenococcus*, *Bacillus*, and *Enterococcus* accounted for more than 80% of the total community. In the fungal community, *Zygosaccharomyces rouxii*, *Candida versatilis*, *Rhodotorula taiwanensis*, *Debaryomyces hansenii*, and *Aspergillus oryzae* were dominant, while the viral community consisted entirely of bacteriophages, with *Bacillus* phages SIOphi accounting for 93%. According to the results of the PLS analysis, desirable sensory characteristics, such as umami, sweet, and roasted soybean, as well as preference, were associated with *Tetragenococcus*, *Lysinibacillus*, *Enterococcus*, *Staphylococcus*, *Lactobacillus*, *Pediococcus*, and *Weissella*. The musty flavor, which is a typical property of traditional fermented foods, was related to *Halomonas* and *Psychrobacte*, while the bitter, acrid taste and sour smell were closely associated with *Chromohalobacter*. The results of this study provide comprehensive information on the microbial community of *ganjang* and may be used to select starter cultures for soy sauces.

## 1. Introduction

**Figure 1:**
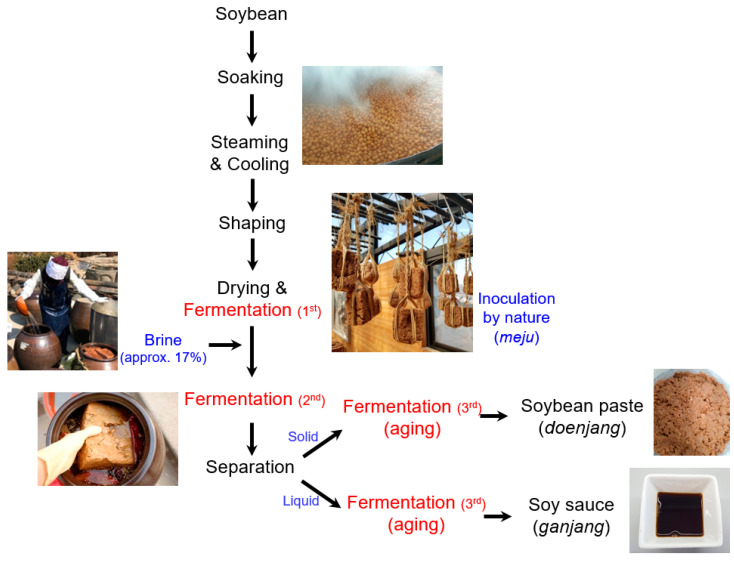
The manufacturing process of Korean traditional fermented soybean foods.

Soy sauce is a liquid seasoning that is produced by fermenting soybeans and has long been consumed, mainly in Asian countries [1]. Korean fermented soy sauce, or *ganjang*, has been an essential condiment of Korean cuisine for many years because it has favorable flavors, including savory and umami. Traditional *ganjang* is produced through a three-stage spontaneous fermentation process using only soybeans, water, and salt. Unlike modernized *ganjang*, which relies on the artificial inoculation of starter cultures (i.e., *Aspergillus oryzae*), traditional *ganjang* does not require a starter culture (Figure 1). The first fermentation takes place in a solid state, resulting in the formation of fermented soybean bricks known as *meju* in Korean. Subsequently, the *meju* are fermented in a ceramic jar filled with brine [approximately 17% (*w*/*v*)] [2]. After the secondary fermentation, they are separated into a liquid part (*ganjang*) and a solid part (soybean paste, or *doenjang*), and each undergoes an additional fermentation (aging) process of at least 6–12 months [3]. The maturation period can last for more than 5 years.

One of the key factors in determining the flavor of *ganjang* is its microbial community. Recently, next-generation sequencing (NGS) technology has been widely used to understand the microbial communities of various fermented foods worldwide [4,5,6,7]. In a previous study, the bacterial composition of Korean traditional fermented soybean paste (*doenjang*) was identified using pyrosequencing, targeting the hyper-variable regions, i.e., V1/V2, of the 16S rRNA gene [8]. Bacterial and fungal community dynamics were also investigated through the sequencing of bacterial 16S rRNA (V1–V3 variable regions) and fungal 28S rRNA (D1–D2 regions) genes [9]. Furthermore, not only the sensory characteristics but also the volatile and non-volatile metabolites that cause the *doenjang* flavors were reported in some previous studies [10,11,12]. Recently, the correlation among the microbial communities, metabolites, and sensory characteristics of *doenjang* was explored [13,14,15]. However, relatively few studies have been conducted on the microbial communities of *ganjang*. Changes in the microbial communities of *ganjang* during a 6-month fermentation period were investigated using amplicon-based community analysis [16,17]. The microbial communities of forty traditional soy sauces collected in South Korea were also analyzed using amplicon-based sequencing [18].

Most of the metagenomic analyses of previous studies were conducted by sequencing the partial region in specific DNA targets such as the 16S-, 18S-, and 26S rRNA or internal transcribed spacer region in order to identify the microorganisms contained in fermented soybean foods. Although these amplicon-based sequencing methods are rapid and economical, they are sensitive to the sequence of the primers used and the number of PCR cycles, which results in low phylogenetic resolution and low discriminatory power, which makes them inaccurate at the species level [19,20,21].

Moreover, information on the relationship between microbial communities and sensory characteristics is also limited. Although the relationship between the bacterial community and sensory characteristics of *doenjang* has already been investigated [15], there has not yet been any similar research on the relationship between the bacterial community and sensory attributes of *ganjang*. In the present study, a shotgun metagenomic approach using Illumina sequencing technology was employed for the analysis of various *ganjang* products. To reveal the microbial communities of *ganjang* as accurately as possible, the entire metagenomes of twenty samples of *ganjang* were sequenced, and a descriptive sensory analysis of *ganjang* was also performed. Finally, the correlation between the sensory characteristics and the microbial community of *ganjang* was investigated by partial least square (PLS) regression analysis.

## 2. Materials and Methods

### 2.1. Soy Sauce Samples

The twenty samples of *ganjang* used in this study are listed in Table S1. Eighteen of the samples (G1–G18) were collected from small regional companies that have been certified as producers of “Traditional Korean Food” by the Ministry of Agricultural, Food, and Rural Affairs of the Republic of Korea. These soy sauces are produced using a traditional method of natural fermentation. Unlike samples G1–G18, however, the other two samples (G19 and G20), which were purchased from a market, are produced using an inoculation of *Aspergillus oryzae* for mass production (modernized method).

### 2.2. Descriptive Sensory Analysis

In particular, 110 semi-trained panelists (Korea Food Research Institute, Wanju, Republic of Korea), who were able to distinguish between five basic tastes (sweet, salty, sour, bitter, and umami) using ten sets of a triangle test [22], were employed to undertake a descriptive sensory analysis of *ganjang*. The *ganjang* samples were stored under refrigeration at 4 °C until the test. Before the experiment, 10 g of each sample was transferred to an opaque polystyrene container and covered with a lid. The samples were randomly labeled with a three-digit number and served to each panelist. To evaluate the smell of each sample, the panelists opened the lid of the container halfway, sniffed the sample three times, and closed the lid immediately. The panelists then opened the lid of each sample container to appraise the appearance of the *ganjang*. The taste and aftertaste of the *ganjang* were estimated by tasting each sample using disposable pipettes and spoons holding two drops of each sample. The twenty samples were assessed in terms of twenty descriptive characteristics relating to preference, appearance, smell, and taste. A nine-point scale ranging from extremely undesirable (1) to desirable (9) was used to evaluate preferences, while a seven-point scale ranging from extremely low (or weak) (1) to high (or strong) (7) was used to evaluate sensory intensities. The panelists were not allowed to drink or eat anything except water during the 2 h before the descriptive sensory analysis. The panelists rinsed their mouths with purified tap water (room temperature) between each sample. The test was carried out in triplicate. Participants gave informed consent via the statement “I am aware that my responses are confidential, and I agree to participate in this survey”, where an affirmative reply was required to enter the survey. They were able to withdraw from the survey at any time without giving a reason. All *ganjang* samples used in this study are products approved for sale in Korea. The products tested were safe for consumption.

### 2.3. Extraction of Metagenomic DNA

To extract the metagenome of the *ganjang*, 10 mL of each of the twenty samples of *ganjang* was centrifuged at 8000× *g* for 30 min. Next, the samples were frozen using liquid nitrogen and then homogenized. To lyse the microbial cells in the samples, STES buffer [0.5 M NaCl, 0.2 M Tris–HCl (pH 7.6), 0.01 M EDTA, 1% SDS] was added to each sample and incubated at 60 °C for 12 h. After the cell lysis, the whole DNA of the samples was extracted using the phenol/chloroform/isoamylalcohol extraction method, and then the RNase was treated. After incubation for 1 h at 37 °C, the whole DNA of each sample was purified using a purification column (iNtRON Biotechnology Co., Ltd., Seongnam, Republic of Korea). The concentration and the purity of the whole DNA were checked through 1% agarose gel electrophoresis using a NanoDrop ND-1000 spectrophotometer (NanoDrop Technologies, Wilmington, DE, USA).

### 2.4. Metagenomic DNA Sequencing

Metagenome sequencing was carried out by Macrogen Inc. (Seoul, Republic of Korea). To cover a wide range of DNA regions, including high GC-rich regions, Illumina libraries were prepared using a TruSeq DNA PCR-free kit (Illumina, San Diego, CA, USA) according to the manufacturer’s instructions. All the libraries were sequenced on an Illumina Hiseq4000 (Illumina, San Diego, CA, USA) instrument using 2 × 10^1^ paired-end reads.

### 2.5. De Novo Assembly of the Sequencing Results

The data processing and analysis were conducted by Seeders Inc. (Daejeon, Republic of Korea). Before assembling the sequencing data, the reads were filtered to eliminate low-quality reads (phred score < 20) and adaptor-only reads (number of nucleotides ≤ 25 bp) using a SolexaQA package program (ver.1.13) [23]. After the pre-processing, the cleaned reads were used for de novo assembly using SOAPdenovo2 (ver.2.04) [24].

### 2.6. Analysis of Microbial Communities and Annotation

To identify the microorganisms, a homology search using BlastN (v2.2.31) with a parameter E-value of ≤1^−10^, an identity of ≥90%, and a query coverage of ≥90% at the NCBI (National Center for Biotechnology Information) (http://www.ncbi.nlm.nih.gov/nuccore, accessed on 2 December 2024) was performed. Microbial abundance was discovered through the alignment of assembled contigs with cleaned reads using Bowtie2 (v2.1.0) software [25].

### 2.7. Statistical Analysis

To investigate the relationship between the microbial communities and the sensory characteristics of the *ganjang* samples, a PLS regression analysis was performed on the relative abundance of microbes and the sensory characteristic values using XLSTAT (Version 2017, Addinsoft, New York, NY, USA). The microbial abundances at the genus level were used as explanatory variables, and the average values of the sensory characteristic data were used as dependent variables. The stop condition was fixed to two components.

## 3. Results

### 3.1. Descriptive Sensory Analysis of Ganjang Samples

The results of the descriptive sensory analysis of the twenty *ganjang* products are shown in Table 1. In terms of appearance, the intensity values of “brown color” and “turbidity” of most samples tended to be proportional to the value of the whole preference. In the analysis of smell and taste characteristics, G19 and G20 (modernized *ganjang* samples) showed high scores for desirable characteristics, including sweet, umami, and roasted soybean, which may be due to the addition of wheat and food additives, such as sweeteners and flavor enhancers (Table S1). It has been reported that the addition of wheat powder in the *meju* production process contributes to enhancing the quality of soy sauce, as wheat has a higher carbohydrate content and a lower protein content than soybeans [26]. Among the traditional soy sauce samples, G3, G6, G10, and G12 exhibited strong roasted bean, sweet, and umami characteristics, which led to high scores for whole preference. It has been reported that amino acids, including alanine, glycine, and serine, generated by proteolysis during fermentation, contribute to sweet taste [27]. It has also been reported that 4-hydroxy-2(or 5)-ethyl-5(or 2)-methyl-3(2*H*) furanone (HEMF) is implicated in the caramel-like flavor of soy sauce [1]. Therefore, the sweet characteristics of the traditional samples generated by fermentation may be different from those of the modernized samples.

### 3.2. Microbial Communities of Ganjang Samples

#### 3.2.1. Overview and Bacterial Community

Most of the previous studies dealing with the microbial community in fermented soybean foods have focused on the genus level or higher due to the limitation of reliability. In this study, the whole metagenome of each of the twenty *ganjang* samples was sequenced in order to identify its microbial communities in more detail and accurately. The results of whole metagenome sequencing showed that bacteria accounted for more than 99% of the microbial community in all of the samples, except G1 (90.0%) and G16 (93.1%) (Figure S1a). At the phylum level, Firmicutes and Proteobacteria accounted for >98% of the majority of ganjang samples, and the Eukaryota kingdom in the G16 sample appeared to belong mostly to the Ascomycota phylum (Figure S1b). At the family level, the microbial communities were much more diverse across the samples (Figure S1c). The predominant families varied across the samples; however, the *Bacillaceae*, *Enterococcaceae*, *Enterobacteriaceae*, and *Leuconostocaceae* families were commonly dominant, either singly or together in all the samples.

**Figure 2:**
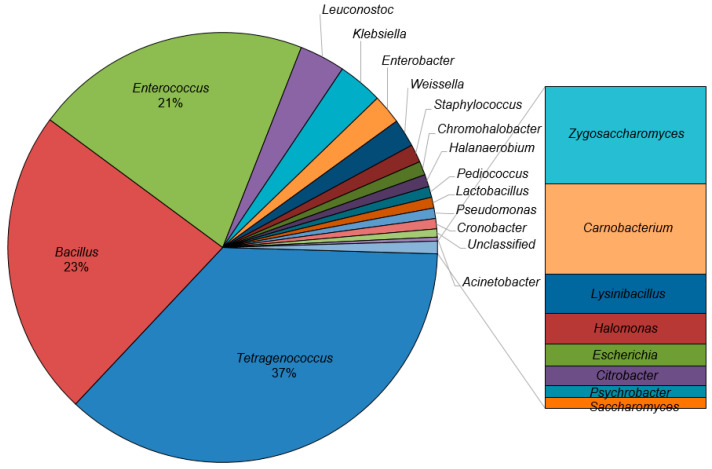
Average microbial composition of twenty samples of *ganjang* according to genus level.

**Figure 3:**
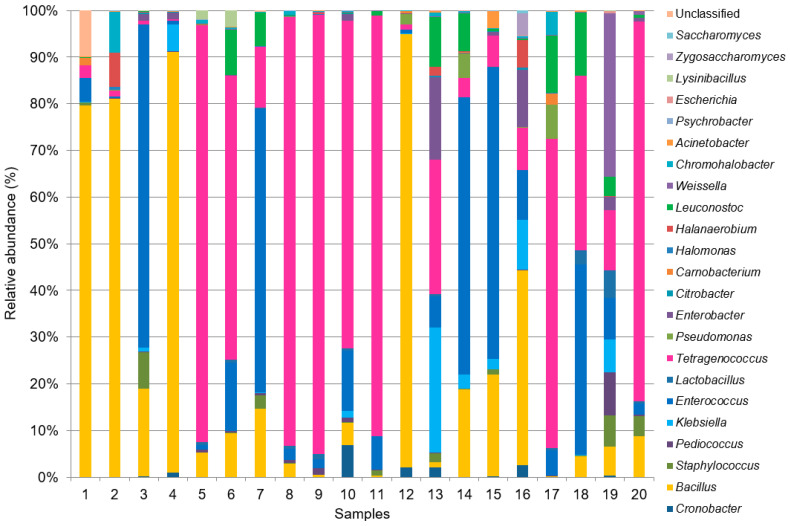
Microbial composition of each *ganjang* sample according to genus level.

At the genus level, a total of 279 genera were observed in all twenty *ganjang* samples, and the top twenty-four genera, accounting for 98.7% of the abundance, are shown in Figure 2. The microbial composition varied among the samples, and there seemed to be no relation with the manufacturing region (Figure 3 and Table S1). In terms of the average of the twenty *ganjang* microbial communities at the genus level, *Tetragenococcus* (37%) was the most dominant, followed by *Bacillus* (23%) and *Enterococcus* (21%) (see Figure 2). These three bacteria genera presented in all of the *ganjang* samples and accounted for approximately 80% of the whole microbial community of the samples, except for G13, G16, and G19 (Figure 2).

In terms of species, the *Tetragenococcus* genus, consisting solely of the species *T. halophilus* (99.9%) and *T. muriaticus* (0.1%), dominated samples G5, G6, G8, G9, G10, G11, G17, and G20 (Figure S2a and Figure 3). *Bacillus* genus, composed of thirty-one species, was the major genus in samples G1, G2, G4, and G12 (Figure 3). Among these, five species (*B. methylotrophicus*, *B. paralicheniformis*, *B. licheniformis*, *B. subtilis*, and *B. amyloliquefaciens*) accounted for more than 94% of the *Bacillus* genus (Figure S2b). Meanwhile, the samples G3, G7, G14, G15, and G18 were dominated by the *Enterococcus* genus (Figure 3), while *E. faecium* accounted for 97.1% of the *Enterococcus* genus (Figure S2c).

With the exception of the three major bacterial genera (*Tetragenococcus*, *Enterococcus*, and *Bacillus*), other microbes accounted for less than 20% of the whole microbial community (Figure 2). Among them, lactic acid bacteria, including *Leuconostoc* (3.4%), *Weissella* (2.1%), *Pediococcus* (0.8%), *Lactobacillus* (0.8%), and *Lysinibacillus* (0.1%), were discovered in the *ganjang* samples. *Leuconostoc* was present in most of the samples, especially in G6, G7, G13, G14, G17, G18, and G19, with a relatively high population (Figure 3). In the *Leuconostoc* population, *L. mesenteroides*, *L. kimchii*, *L. citreum*, *L. carnosum,* and *L. gelidum* were found, of which 96% were *L. mesenteroides*. In the case of the *Weissella* genus composed of *W. cibaria*, *W. confusa*, *W. koreensis*, *W. thailandensis,* and *W. paramesenteroides*, *W. cibaria* accounted for 80% of the population (Figure S3a). *Weissella* was found at less than 1% abundance in most samples, and it was found in great abundance only in G19 (34.2%) (Figure 3). In the population of *Pediococcus* and *Lactobacillus*, the proportions of *P. pentosaceus* (93.5% of the *Pediococcus* genus) and *L. sakei* (50% of the *Lactobacillus* genus) were the highest in each genus (Figure S3c). 

In addition to lactic acid bacteria, various minor bacteria genera including *Klebsiella* (3.3%), *Enterobacter* (2.2%), *Staphylococcus* (1.3%), *Chromohalobacter* (1.0%), *Halanaerobium* (0.9%), *Pseudomonas* (0.8%), *Cronobacter* (0.8%), *Acinetobacter* (0.3%), *Carnobacterium* (0.3%), *Halomonas* (0.1%), *Escherichia* (0.06%), *Citrobacter* (0.06%), and *Psychrobacter* (0.03%) were also observed (Figure 2). In the *Staphylococcus* genus, *S. saprophyticus*, *S. xylosus*, and *S. sciuri* accounted for more than 70% (Figure S3b). Meanwhile, *Chromohalobacter salexigens* (>99%), *Halanaerobium praevalens* (>96%), and *Pseudomonas psychrophila* (>72%) were present in the highest proportions within their respective genera.

#### 3.2.2. Fungal Community

**Figure 4:**
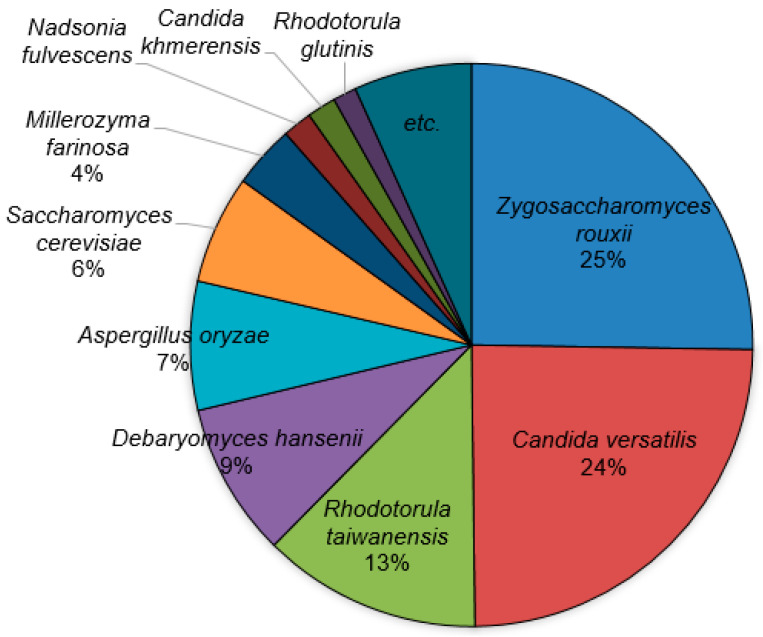
Average fungal composition of nineteen *ganjang* samples according to species level.

Although eukaryotes are generally present in much lower abundance than bacteria in soy sauce, they have been reported to play a key role in generating flavors [16,28,29]. According to the results of an analysis of the microbial community at the superkingdom level, Eukaryota accounted for less than 1% in all samples, except G16 (Figure S1). The analysis of the eukaryotic community in all samples revealed that forty-five of the fifty-four species of Eukaryota were yeasts and molds. Interestingly, the average fungal population in nineteen samples was 7244, while approximately 1.41 million populations were detected in the G16 sample. Therefore, the average fungal community was calculated, excluding the G16 sample, to ensure the representativeness of the *ganjang* fungal community. Among the fungal species, *Zygosaccharomyces rouxii* was most dominant, followed by *Candida versatilis*, *Rhodotorula taiwanensis*, *Debaryomyces hansenii*, *Aspergillus oryzae*, *Saccharomyces cerevisiae*, and *Millerozyma farinosa* (Figure 4).

#### 3.2.3. Viral Community

**Figure 5:**
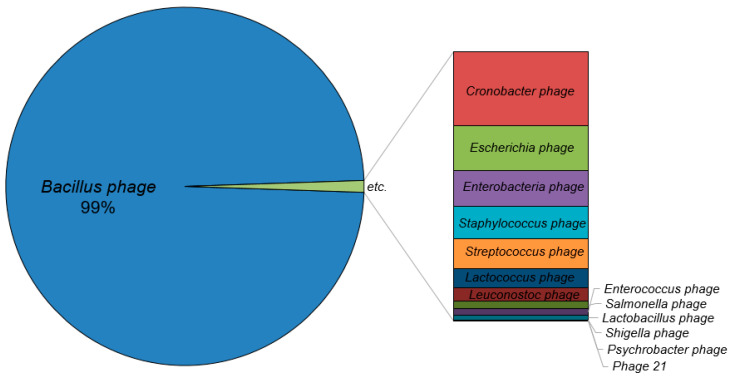
Average viral composition of twenty samples of *ganjang*.

The viral community of the twenty *ganjang* metagenomes was composed of two orders (Caudovirales and Tubulavirales), six families (*Siphoviridae*, *Myoviridae*, *Herelleviridae*, *Podoviridae*, *Peduoviridae*, and *Inoviridae*), eighteen genera, and fifty-seven species. All virus species were bacteriophages, and *Bacillus* phages accounted for 98.6% of the virus population in all the *ganjang* samples (Figure 5). The top nine species were all *Bacillus* phages (SIOphi, phi105, SPG24, SPbeta, phiNIT1, Grass, PM1, BCP8-2, and CampHawk), among which *Bacillus* phage SIOphi accounted for 93%. Interestingly, there were few bacteriophages infecting *Tetragenococcus* and *Enterococcus*, which were the dominant bacteria in all the samples.

### 3.3. Correlation Between Microbial Communities and Sensory Characteristics

**Figure 6:**
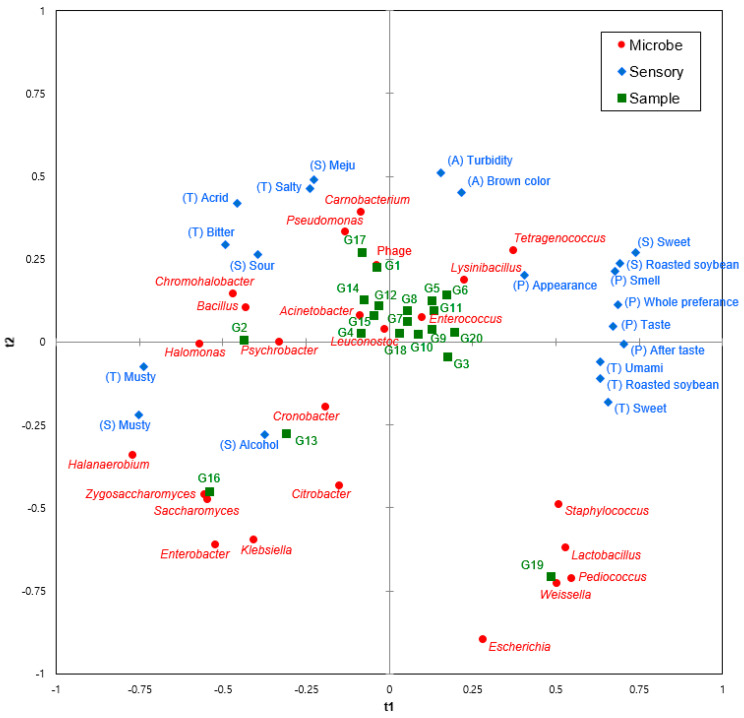
Correlation map between microbial communities and sensory characteristics of *ganjang* samples.

To elucidate the relationship between the sensory characteristics and microbial communities of *ganjang*, a PLS regression analysis was performed using the relative abundance of the dominant microbes and the sensory characteristic scores (Figure 6). The majority of the samples were found in the middle of the correlation map. Based on the positioning of their features, these samples are likely to have indistinct sensory qualities. Samples or sensory characteristics near the middle of the correlation map are considered to have little influence on the analysis [30].

On the other hand, samples G2, G13, G16, and G19 showed significantly different characteristics from the other samples. Samples G2, G13, and G16, located on the negative axis of t1, showed correlations with alcohol and musty flavors. The alcohol flavor may be related to *Zygosaccharomyces* and *Saccharomyces*. These yeasts contribute significantly to the generation of ethanol and higher alcohols (isoamyl alcohol, isobutyl alcohol, and 2-phenylethyl alcohol), which are essential for the distinctive flavor of soy sauce [31]. The musty flavor, which is a typical odor of traditional fermented foods, might be related to *Halomonas* and *Psychrobacter*. *Halomonas* has been reported to be associated with ethyl palmitate, 1-octanol, heptanol, and 2-nonanol, which contribute to the unique odor of *longgang*, a traditional Chinese soy sauce [32]. It has also been reported that lipase produced by *Psychrobacter* can generate a foul, moldy odor by hydrolyzing oils [33,34]. Bitter, acrid tastes and a sour smell were found to be closely related to *Chromohalobacter*. In fact, *Chromohalobacter* has been reported to be responsible for the formation of lactic acid, acetic acid (sour), and putrescine (unpleasant odor, bitter taste) in *ganjang* [35].

Desirable characteristics, such as sweet, umami, and roasted soybean, as well as preference, were all located on the positive axis of t1, and the microorganism with the strongest correlation with them was *Tetragenococcus*. As mentioned above, *T. halophilus* has been reported to play a crucial role in generating the flavor of soy sauce because it has a strong positive correlation with aspartic acid, glutamic acid, and *N*-succinyl-glutamic acid, all of which exhibit the umami flavor [36], as well as volatile compounds with fruity and floral aromas [37]. In addition, *Lysinibacillus*, *Enterococcus*, *Staphylococcus*, *Lactobacillus*, *Pediococcus*, and *Weissella* also showed high correlations with the desirable features. *Staphylococcus* is one of the dominant strains during the fermentation of soy sauce and has been reported to enhance the major fruity flavor compounds of soy sauce, such as ethyl acetate, 3-methyl-1-butanol, 2-methylbutanol, 3-methylbutyraldehyde, and ethyl lactate [38]. *Pediococcus* has been reported to be correlated with 4-ethyl-2-methoxyphenol (bacon and condiment smell) and 2-methoxyphenol (smoky and peaty flavor), while *Lactobacillus* has been reported to be closely correlated with volatile compounds such as maltol (flavor enhancer), ethyl lactate (fruity and buttery), 3-phenylfuran (caramel), and methional (potato snacks) [39].

## 4. Discussion

The microbial composition of traditional Korean soy sauce revealed in this study was somewhat different from that of soy sauces from other countries reported in previous studies. While *Tetragenococcus*, *Bacillus*, and *Enterococcus* were the major bacterial strains in traditional Korean soy sauce, *Tetragenococcus*, *Weissela*, and *Staphylococcus* were the dominant strains in Japanese and Chinese (Cantonese) style soy sauces [29,37]. It may be the case that *Bacillus* is abundant in traditional Korean soy sauce because the bacteria is a major population of *meju* [9]. The manufacturing process of traditional Korean soy sauce involves a unique process of tying the blocks of *meju* with rice straw and hanging them from the ceiling for fermentation, and it is possible that *Bacillus* is derived from rice straw during this process (Figure 1) [16,40]. *Bacillus* is one of the most important bacteria in fermented soybean foods because its strong proteolytic activity contributes to generating the umami flavor [41]. This property also allows *Bacillus* to be used for the industrial production of food-grade endoproteases [42].

*Tetragenococcus* was the most dominant genus on average in the *ganjang* samples analyzed in this study (Figure 2). *T. halophilus* and *T. muriaticus* are halophilic lactic acid bacteria observed in diverse fermented and salted foods, including fermented soybean foods and fish sauces [43]. *T. halophilus* is rarely found in *meju*, and it is reported to be derived from solar salt because it is the main microbe of solar salt [16,17]. *T. halophilus* has been reported to play a pivotal role in generating the flavor of soy sauce because it has a strong positive correlation with aspartic acid, glutamic acid, and *N*-succinyl-glutamic acid, all of which exhibit the umami taste [36], as well as volatile compounds with fruity and floral aromas [37]. Moreover, *T. halophilus* has been reported to be involved in the formation of organic acids, such as lactic acid and acetic acid [29,39]. *T. muriaticus* has been reported to significantly increase the variety and abundance of desirable aldehydes, esters, and alcohols in low-salt fish sauces [44].

*Enterococcus* was the third most abundant genus in the average microbial population (Figure 2). Although *Enterococcus* spp., such as *E. faecalis*, *E. faecium,* and *E. casseliflavus*, are known to be opportunistic pathogens, it has been reported to be one of the most abundant bacteria in various fermented foods including fermented soybean foods and dairy products [8,45]. Although there is some controversy regarding the safety of *Enterococcus*, it has been reported that eighty-eight strains of *E. faecium* isolated from *meju* had no resistance to seven types of antibiotics, including vancomycin, and that they also had no hemolytic activity [46]. *Enterococcus* spp. contributes to the unique flavors of many types of fermented foods since it has high glycolytic, proteolytic, and lipolytic activities [47].

Unlike *meju* and *doenjang*, the presence of lactic acid bacteria has rarely been reported in *ganjang*, and little research has been performed on their role in the generation of soy sauce’s flavor; however, these can be estimated through previous studies on soy sauce from other countries. Lactic acid bacteria such as *Leuconostoc*, *Weissella*, and *Lactobacillus* have been reported to contribute to the formation of organic acids and volatile compounds in soy sauce. In particular, *Weissella* and malic acid [29], *Lactobacillus* and succinic acid, and *Leuconostoc* and flavor volatiles [39] were found to be strongly positively correlated in a metagenomic and metabolomic analysis of Chinese soy sauce.

*Staphylococcus* has been reported to be mainly responsible for succinic acid synthesis, and it also plays a role in nitrite/nitrate degradation [29]. Halophiles such as *Chromohalobacter* and *Halomonas* are less abundant in soy sauce than *Bacillus*, but they are metabolically more active and may play a critical role in the decomposition of soybean components, including starch, cellulose, proteins, and lipids [16]. *Chromohalobacter*, *Halomonas*, and *Staphylococcus* are also dominant bacteria in solar salt, along with *Tetragenococcus*, and might have derived from solar salt [16].

In the case of the fungal community, *Z. rouxii* was the most dominant, followed by *C. versatilis*, *R. taiwanensis*, *D. hansenii*, *A. oryzae*, *S. cerevisiae*, and *M. farinosa* (Figure 4). *Z. rouxii*, along with *C. versatilis*, has been reported to be the most dominant yeast in soy sauce, and it has been widely used as a starter for soy sauce because it produces key caramel-like flavor compounds, such as HEMF and 4-hydroxy-2,5-dimethyl-3 [2H]-furanone (HDMF) [31,48,49,50,51,52]. *C. versatilis* has been reported as a major contributor to the smoky flavor in soy sauce by producing 4-ethylphenol and 4-ethylguaiacol [53]. *D. hansenii* is also reported to be the main yeast for cheese and produces various flavor compounds. In particular, it has an excellent ability to convert ferulic acid into 4-vinylguaiacol, thus contributing to the smoky flavor of fermented soy foods [54]. *Aspergillus*, also known as “*koji*” in Japan and China, is the most important starter used in industrially produced fermented soybean foods. It has been reported that it may not play a significant role in soy sauce fermentation [16], although it is the main fungal group in *meju* [9].

In the case of the viral community, *Bacillus* phages accounted for 98.6% of the virus population in the *ganjang* samples (Figure 5). Cheon et al. have reported that *Bacillus* is metabolically inactive during *ganjang* fermentation [16], which may be related to *Bacillus* phage. Meanwhile, although there have been reports that several pathogenic viruses have been found in fermented foods [55], no viruses other than bacteriophages have been found in the soy sauces investigated in this study. Bacteriophages have been found to significantly influence the quality of fermented foods by directly altering the microbial community composition [55,56,57,58,59]. However, additional studies are required to explore the effects of bacteriophages on soybean fermentation.

## 5. Conclusions

Because traditional Korean fermented soy foods rely on natural fermentation, their microbial communities are very complex, which greatly affect their sensory characteristics. In this study, shotgun metagenomic sequencing was used to discover large-scale microbial communities of Korean soy sauce (*ganjang*) at the species level that were difficult to identify using amplicon-based sequencing in previous studies. In addition, correlation analysis between microbial communities and sensory characteristics identified microorganisms with high correlations with major sensory characteristics of *ganjang*. The current goal of the soy sauce industry is to inoculate a few microbial starters to enhance specific flavors; however, it is expected that technology will be developed in the future to control sensory characteristics more precisely, and the results of this study will serve as the basis for such technology.

## Figures and Tables

**Table 1:** Summary of consumer preference and sensory characteristics of twenty *ganjang* samples.

	G1	G2	G3	G4	G5	G6	G7	G8	G9	G10	G11	G12	G13	G14	G15	G16	G17	G18	G19	G20
(P) Appearance	5.7 ^defg^	4.6 ^h^	6.3 ^abc^	4.1 ^i^	5.5 ^efg^	6.4 ^ab^	4.9 ^g^	6.2 ^abcd^	4.8 ^g^	6.0 ^bcde^	5.9 ^cdef^	6.6 ^a^	5.8 ^defg^	5.4 ^g^	5.6 ^efg^	4.7 ^h^	5.7 ^efg^	4.5 ^hi^	5.5 ^fg^	6.7 ^a^
(P) Smell	5.3 ^bcd^	3.9 ^gh^	6.0 ^a^	3.5 ^h^	4.8 ^def^	6.1 ^a^	4.3 ^fg^	5.1 ^cde^	4.4 ^fg^	5.4 ^bc^	4.5 ^f^	5.7 ^ab^	3.0 ^i^	3.9 ^gh^	4.7 ^ef^	3.4 ^hi^	4.8 ^def^	3.9 ^gh^	5.4 ^bc^	5.7 ^ab^
(P) Taste	4.7 ^fg^	4.3 ^g^	6.1 ^bc^	3.7 ^h^	5.3 ^de^	6.3 ^ab^	4.7 ^fg^	5.1 ^ef^	5.4 ^de^	5.7 ^cd^	4.7 ^fg^	6.1 ^bc^	3.3 ^h^	4.2 ^g^	4.7 ^fg^	4.3 ^g^	5.0 ^ef^	4.2 ^g^	6.0 ^bc^	6.7 ^a^
(P) Aftertaste	4.5 ^ghi^	4.2 ^hij^	5.8 ^abc^	3.7 ^j^	5.0 ^efg^	6.1 ^a^	4.4 ^ghi^	4.8 ^fgh^	5.1 ^def^	5.4 ^cde^	4.4 ^ghi^	5.5 ^bcd^	3.1 ^k^	4.0 ^ij^	4.6 ^ghi^	4.1 ^ij^	4.7 ^fgh^	4.1 ^ij^	5.9 ^ab^	6.1 ^a^
(P) Whole preference	4.7 ^ghi^	4.2 ^jk^	6.0 ^bc^	3.6 ^l^	5.4 ^def^	6.2 ^ab^	4.6 ^ghij^	5.3 ^ef^	5.1 ^fg^	5.6 ^cde^	4.8 ^gh^	6.1 ^abc^	3.3 ^l^	4.4 ^hijk^	4.7 ^ghi^	4.1 ^k^	5.0 ^fg^	4.2 ^ijk^	5.8 ^bcd^	6.6 ^a^
(A) Brown color	4.7 ^f^	3.6 ^h^	6.3 ^a^	2.5 ^i^	4.3 ^g^	5.9 ^b^	3.8 ^h^	5.0 ^de^	2.0 ^j^	5.9 ^b^	5.2 ^cd^	5.2 ^cd^	4.9 ^ef^	3.5 ^h^	4.6 ^fg^	2.1 ^j^	5.4 ^c^	2.7 ^i^	2.5 ^i^	5.5 ^c^
(A) Turbidity	4.5 ^de^	3.6 ^fg^	5.8 ^a^	2.7 ^h^	4.1 ^e^	5.1 ^b^	3.7 ^f^	4.4 ^de^	2.0 ^i^	5.2 ^b^	4.6 ^cd^	4.5 ^de^	4.3 ^de^	3.3 ^g^	4.3 ^de^	2.1 ^i^	4.9 ^bc^	2.8 ^h^	2.2 ^i^	4.5 ^de^
(S) Roasted soybean	3.7 ^defg^	3.1 ^ij^	4.3 ^ab^	3.0 ^j^	4.0 ^bcde^	4.4 ^a^	3.6 ^efg^	4.3 ^ab^	4.1 ^abc^	4.0 ^abcd^	3.4 ^ghi^	4.3 ^ab^	3.1 ^hij^	3.5 f^gh^	3.6 ^defg^	3.0 ^j^	3.7 ^cdefg^	3.4 ^ghij^	3.9 ^bcdef^	4.4 ^a^
(S) Sweet	3.6 ^ef^	3.2 ^ghi^	4.4 ^ab^	3.2 ^ghi^	3.8 ^de^	4.5 ^a^	3.6 ^ef^	4.1 ^bcd^	3.9 ^cde^	4.0 ^cde^	3.7 ^def^	4.2 ^abc^	3.0 ^hi^	3.5 ^efg^	3.8 ^cde^	2.9 ^i^	3.8 ^def^	3.4 ^fgh^	4.0 ^cde^	4.6 ^a^
(S) Sour	3.8 ^abc^	3.7 ^abcd^	3.5 ^bcde^	3.9 ^ab^	3.8 ^abc^	3.3 ^cdef^	3.8 ^abc^	3.1 ^ef^	3.0 ^f^	3.5 ^bcde^	3.8 ^abc^	3.5 ^bcde^	3.5 ^bcde^	4.0 ^a^	3.7 ^abcd^	3.7 ^abcd^	3.6 ^abcd^	3.7 ^abc^	3.2 ^def^	3.5 ^bcde^
(S) Meju	4.7 ^cde^	4.8 ^bcde^	4.0 ^gh^	4.4 ^defg^	5.2 ^ab^	4.4 ^efg^	4.8 ^abcd^	4.8 ^bcde^	5.2 ^ab^	4.4 ^defg^	4.5 ^def^	4.8 ^bcde^	4.8 ^bcde^	5.0 ^abc^	4.2 ^fgh^	4.2 ^fgh^	5.0 ^abc^	5.3 ^a^	3.9 ^h^	3.9 ^h^
(S) Alcohol	3.4 ^bcd^	3.3 ^cde^	3.2 ^cde^	3.5 ^bcd^	3.5 ^bc^	3.2 ^cde^	3.5 ^bc^	3.0 ^de^	2.9 ^e^	3.5 ^bc^	3.3 ^cde^	3.2 ^cde^	3.6 ^bc^	3.9 ^ab^	3.4 ^bcd^	4.0 ^a^	3.2 ^cde^	3.3 ^cde^	3.3 ^cde^	3.8 ^ab^
(S) Musty	3.4 ^ghij^	4.3 ^bc^	3.0 ^j^	4.2 ^cd^	3.8 ^defg^	3.0 ^j^	3.8 ^defg^	3.4 ^ghij^	3.6 ^fghi^	3.4 ^ghij^	4.0 ^cdef^	3.1 ^ij^	5.2 ^a^	4.2 ^cd^	3.6 ^fghi^	4.6 ^b^	3.7 ^efgh^	4.1 ^cde^	3.1 ^ij^	3.2 ^hij^
(T) Sweet	3.2 ^hi^	3.3 ^hi^	4.1 ^cd^	3.2 ^i^	3.9 ^cde^	4.2 ^bc^	3.5 ^fghi^	3.7 ^defg^	3.9 ^cdef^	4.1 ^cd^	3.4 ^ghi^	4.2 ^bc^	3.4 ^ghi^	3.4 ^ghi^	3.4 ^ghi^	3.4 ^ghi^	3.6 ^efgh^	3.4 ^ghi^	4.5 ^b^	5.0 ^a^
(T) Umami	3.9 ^fg^	3.7 ^gh^	4.5 ^bcd^	3.4 ^h^	4.5 ^bcd^	4.6 ^bc^	4.0 ^efg^	4.2 ^def^	4.3 ^cde^	4.5 ^bcd^	3.9 ^efg^	4.9 ^ab^	3.7 ^gh^	3.7 ^gh^	4.0 ^efg^	3.8 ^fg^	4.0 ^efg^	3.7 ^gh^	4.7 ^bc^	5.1 ^a^
(T) Roasted soybean	3.3 ^fg^	3.1 ^fg^	3.9 ^bc^	3.0 ^g^	3.7 ^cde^	4.1 ^bc^	3.4 ^def^	3.8 ^bcd^	3.8 ^bcd^	3.8 ^bcd^	3.4 ^efg^	4.1 ^b^	3.3 ^efg^	3.3 ^fg^	3.4 ^def^	3.3 ^fg^	3.4 ^def^	3.1 ^fg^	4.1 ^bc^	4.5 ^a^
(T) Salty	5.6 ^a^	5.3 ^ab^	5.2 ^abc^	5.4 ^ab^	5.4 ^ab^	5.3 ^abc^	5.5 ^a^	5.4 ^ab^	5.0 ^bcd^	5.3 ^ab^	5.5 ^a^	5.3 ^ab^	4.9 ^cd^	5.1 ^bcd^	5.3 ^ab^	5.2 ^abc^	5.4 ^ab^	5.4 ^ab^	4.8 ^d^	4.3 ^e^
(T) Astringent	3.8 ^ab^	3.5 ^bc^	3.2 ^cd^	3.8 ^ab^	3.8 ^ab^	3.0 ^de^	3.4 ^bc^	3.8 ^ab^	3.4 ^bcd^	3.2 ^cd^	4.0 ^a^	3.2 ^cd^	3.8 ^ab^	3.9 ^ab^	3.6 ^abc^	3.5 ^abc^	3.8 ^ab^	3.9 ^ab^	2.7 ^e^	2.7 ^e^
(T) Bitter	3.6 ^abc^	3.4 ^abcd^	3.0 ^def^	3.6 ^ab^	3.3 ^bcd^	2.8 ^efg^	3.2 ^bcde^	3.3 ^abcd^	2.8 ^efg^	3.2 ^bcde^	3.7 ^a^	2.8 ^egf^	3.8 ^a^	3.8 ^a^	3.4 ^abcd^	3.1 ^cde^	3.4 ^abcd^	3.4 ^abcd^	2.6 ^fg^	2.5 ^g^
(T) Musty	3.4 ^defg^	3.8 ^bc^	2.7 ^ijk^	4.1 ^b^	3.2 ^fghi^	2.4 ^k^	3.3 ^fgh^	3.3 ^efg^	3.0 ^ghij^	2.8 ^hijk^	3.4 ^defg^	2.6 ^jk^	4.9 ^a^	3.8 ^bcde^	3.4 ^dfg^	3.8 ^bcd^	3.3 ^fg^	3.5 ^cdef^	2.4 ^k^	2.4 ^k^

Different superscripts within a row mean significant differences at *p* < 0.05. Abbreviations are as follows: (P), preference; (A), appearance; (S), smell; (T), taste.

## Data Availability

The original contributions presented in this study are included in the article/supplementary material. Further inquiries can be directed to the corresponding author.

## Supplementary Materials

The following supporting information can be downloaded at https://www.mdpi.com/article/10.3390/microorganisms12122559/s1, Figure S1: Microbial communities of twenty samples of *ganjang* at the superkingdom (a), phylum (b), and family (c) levels; Figure S2: Species composition of the three major genera [*Tetragenococcus* (a), *Bacillus* (b), and *Enterococcus* (c)] in twenty *ganjang* samples; Figure S3: Species composition of minor genera [excluding genera dominated (>90%) by one species] [*Weissella* (a), *Staphylococcus* (b), and *Lactobacillus* (c)] in twenty *ganjang* samples; Table S1: Information on the twenty samples of *ganjang* used in this research.
