# Supplementary material for: Investigation of Microbial Community of Korean Soy Sauce (Ganjang) Using Shotgun Metagenomic Sequencing and Its Relationship with Sensory Characteristics
Source: Microorganisms. 2024 Dec 12;12(12):2559. doi: 10.3390/microorganisms12122559 (PMC11678514; doi:10.3390/microorganisms12122559)

Table S1. Information on the twenty samples of *ganjang* used in this research

Samples	Region	Ingredient and content	Salt contents (%)
G1	Gangwon (Northeast of Korea)	Meju, salt water	29.89±0.06
G2		Meju, salt	26.03±0.29
G3	Gyeonggi (Northwest of Korea)	Meju, salt, purified water	27.79±0.29
G4		Soybean, salt, purified water	26.85±0.06
G5		Purified water, Korea style meju, solar salt	26.79±0.12
G6	Chungcheong (Middle of Korea)	Meju, purified water, solar salt	27.96±0.12
G7		Meju, solar salt	27.90±0.18
G8	Gyeongsang (Southeast of Korea)	Soybean, refined salt, purified water	23.22±0.18
G9		Korea style meju, refined salt, purified water	18.49±0.23
G10		Soybean, solar salt	29.57±0.29
G11	Jeolla (Southwest of Korea)	Meju, salt water	22.52±0.29
G12		Meju, salt water	25.45±0.06
G13		Soybean, solar salt, purified water	17.78±0.12
G14		Soybean, solar salt, purified water	17.67±0.12
G15		Meju, solar salt	26.33±0.00
G16	Jeju island	Korea style meju, purified water, solar salt	23.46±.060

G17		Soybean, solar salt, water	26.21±0.12
G18		Soybean, solar salt, water	21.99±0.18
G19	Modernized *ganjang*	Meju (soybean, refined salt, koji), solar salt, ethyl alcohol, yeast extract, kelp extract, garlic concentrate, flavour enhancer, nutrient fortification	16.91±0.06
G20		Refined water, defatted soybean, wheat, solar salt, high fructose corn syrup, ethyl alcohol, koji, enzymatically modified stevia glucosyl stevia, yeast extract, licorice extract	23.99±0.00

Figure S1. Microbial communities of twenty samples of *ganjang* at superkingdoam (a), phylum (b), and family (c) levels

(a) Superkingdom

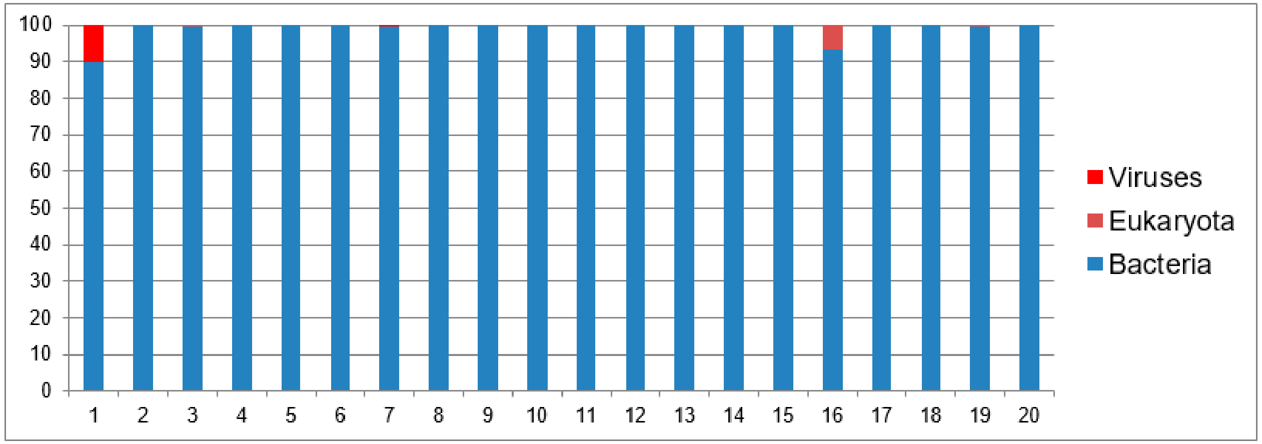

(b) Phylum

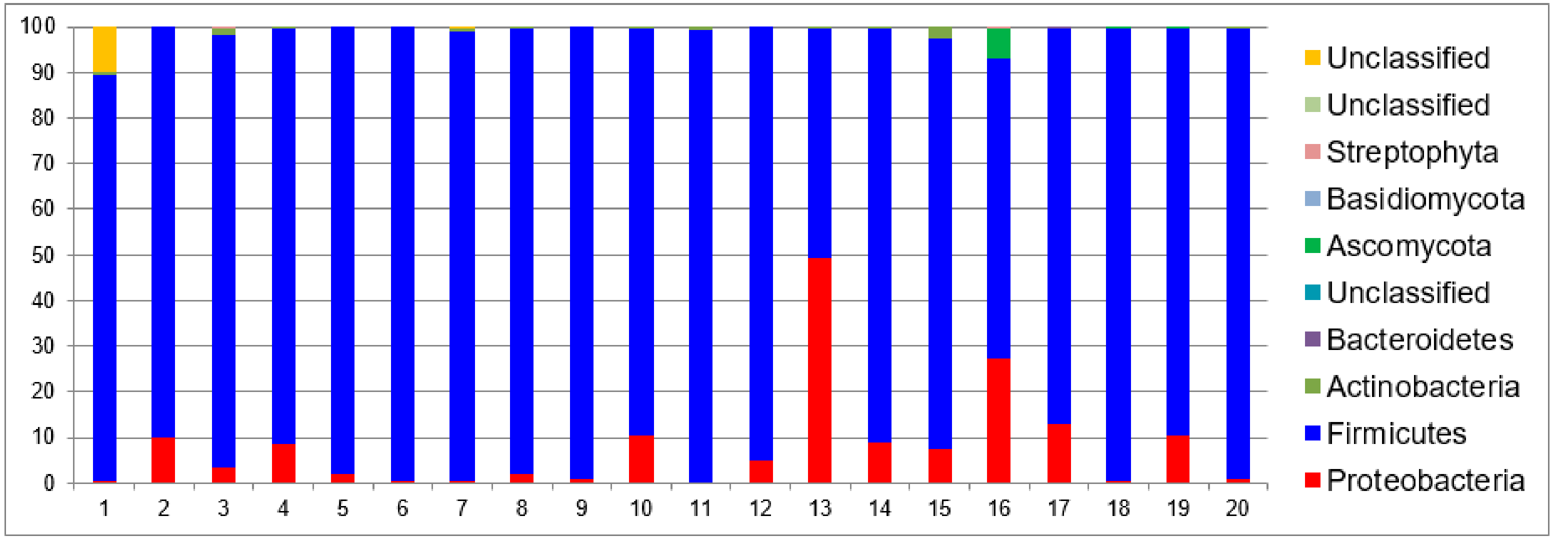

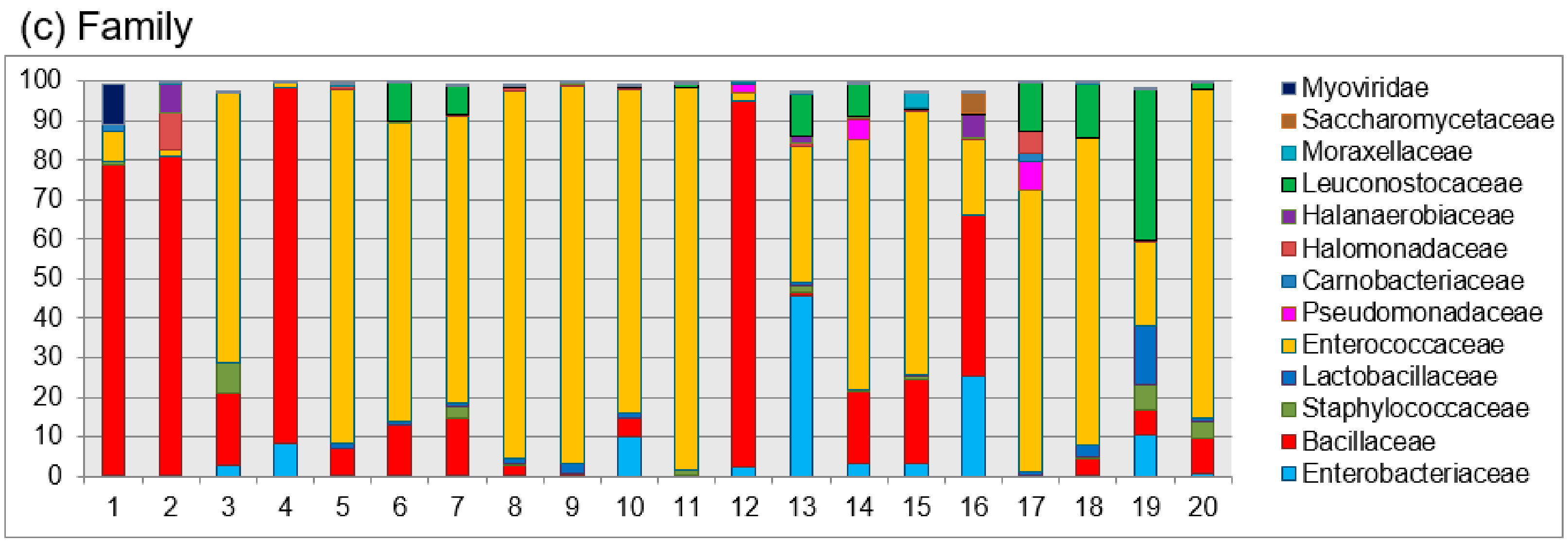

(c) Family
100
90
80
70
60
50
40
30
20
10
0
1
2
3
4
5
6
7
8
9
10
11
12
13
14
15
16
17
18
19
20
Myoviridae
Saccharomycetaceae
Moraxellaceae
Leuconostocaceae
Halanaerobiaceae
Halomonadaceae
Carnobacteriaceae
Pseudomonadaceae
Enterococcaceae
Lactobacillaceae
Staphylococcaceae
Bacillaceae
Enterobacteriaceae

Figure S2. Species composition of the three major genera [*Tetragenococcus* (a), *Bacillus* (b), and *Enterococcus* (c)] in twenty *ganjang* samples

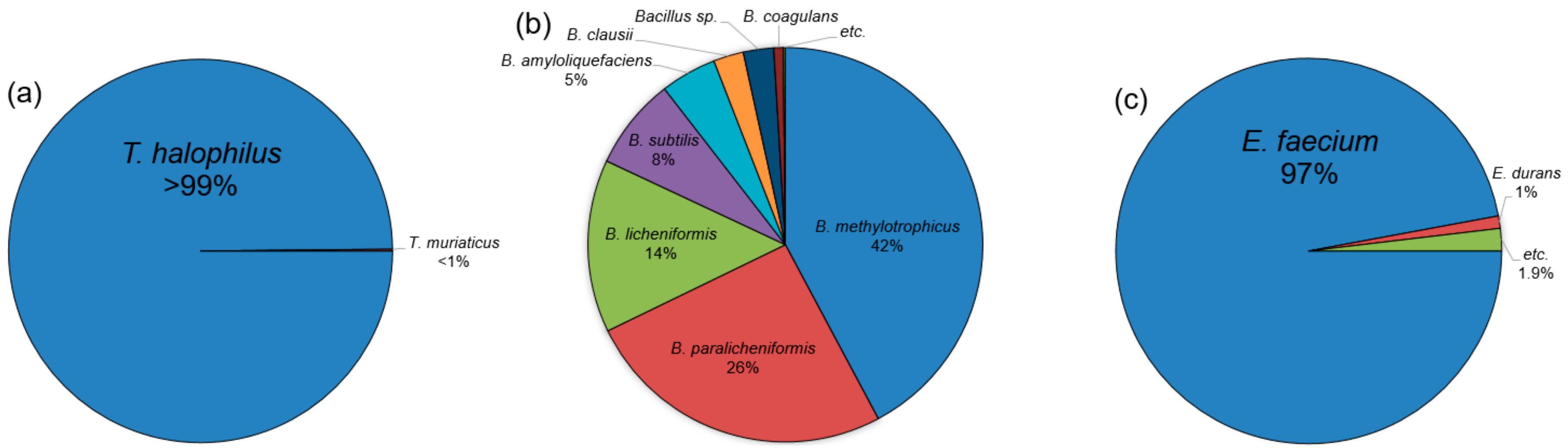

Figure S3. Species composition of minor genera [excluding genera dominated (>90%) by one species] [*Weissella* (a), *Staphylococcus* (b), and *Lactobacillus* (c)] in twenty *ganjang* samples

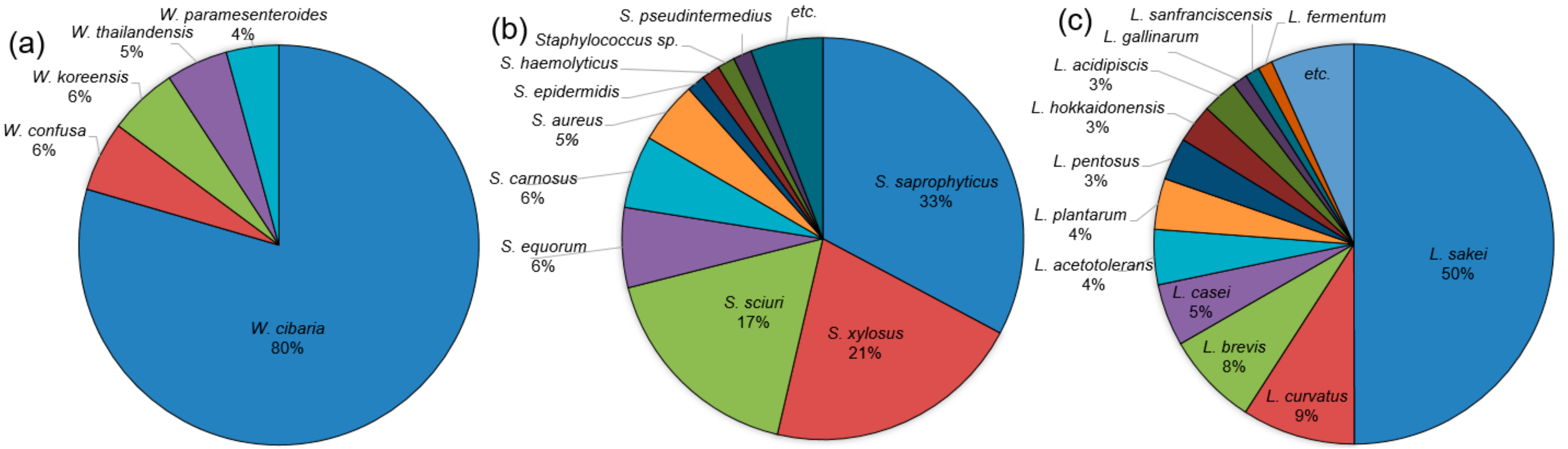

Supplement: Supplementary file 1 [file microorganisms-12-02559-s001.zip › microorganisms-3379569-supplementary.pdf]
